# Morphological Clues of Acute Monocytic Leukemia in COVID-19-Induced Transient Leukoerythroblastic Reaction with Monocytosis

**DOI:** 10.3390/hematolrep16020033

**Published:** 2024-05-28

**Authors:** Ingrid S. Tam, Mohamed Elemary, John DeCoteau, Anna Porwit, Emina E. Torlakovic

**Affiliations:** 1 Department of Pathology and Laboratory Medicine, College of Medicine, University of Saskatchewan, Saskatoon, SK S7N 5A2, Canada; ingrid.tam@usask.ca (I.S.T.); john.decoteau@usask.ca (J.D.); 2 Saskatchewan Cancer Agency, Saskatoon, SK S4W 0G3, Canada; mohamed.elemary@saskcancer.ca; 3 Faculty of Medicine, Department of Clinical Sciences, Division of Oncology and Pathology, Lund University, 221 00 Lund, Sweden; anna.porwit@med.lu.se; 4 Saskatchewan Health Authority (SHA), Saskatoon, SK S7K 0M7, Canada

**Keywords:** acute myeloid leukemia, COVID-19, monocytes, promonocytes

## Abstract

Viral infections, including those caused by COVID-19, can produce striking morphologic changes in peripheral blood. Distinguishing between reactive changes and abnormal morphology of monocytes remains particularly difficult, with low consensus rates reported amongst hematopathologists. Here, we report a patient who developed transient monocytosis of 11.06 × 10^9^/L with 32% promonocytes and 1% blasts during hospitalization that was secondary to severe COVID-19 infection. Three days later, the clinical status of the patient improved and the WBC had decreased to 8.47 × 10^9^/L with 2.2 × 10^9^/L monocytes. Flow cytometry studies did not reveal immunophenotypic findings specific for an overt malignant population. At no time during admission did the patient develop cytopenia(s), and she was discharged upon clinical improvement. However, the peripheral blood sample containing promonocytes was sent for molecular testing with an extended next-generation sequencing myeloid panel and was positive for pathogenic *NPM1* Type A and *DNMT3A* R882H mutations. Subsequently, despite an essentially normal complete blood count, the patient underwent a bone marrow assessment that showed acute myeloid leukemia with 77% promonocytes. This case emphasizes the critical importance of a full work up to exclude acute leukemia when classical promonocyte morphology is encountered in the peripheral blood. Promonocytes are not a part of the reactive changes associated with COVID-19 and remain specific to myeloid neoplasia.

## 1. Introduction

COVID-19 infection can result in morphological changes in the peripheral blood involving lymphocytes, monocytes, and granulocytes [1,2,3,4]. Although most of these changes are non-specific, some changes in monocytes have been described as characteristic of COVID-19. In general, reactive monocytes may show rather diverse morphologies that can affect the nuclear/cytoplasmic ratio, cell size, nucleus size, chromatin changes, and almost universally cytoplasmic changes [5]; distinguishing normal from abnormal monocytes can be particularly challenging [6]. There is one type of monocyte morphology, namely promonocytes, that is always considered to be diagnostic of immature monocytes, equivalent to blasts, and is seen only in some myeloid neoplasms, especially in acute monocytic leukemia [7]. As we continue to learn about the effect of COVID-19 on peripheral blood morphology, the question of whether the presence of promonocytes may be explained by the cytopathic effect of COVID-19 on monocytes or whether this important cell morphology is still diagnostic of a neoplastic process has arisen. Essentially, the key question is can COVID-19 produce reactive monocytes with promonocyte-like morphology. Our case report describes the sudden surge of monocytosis with a subpopulation of promonocytes in a COVID-19 patient with worsening clinical status and their disappearance following quick clinical improvement when there was no evidence of cytopenia at any time before, during, or after hospitalization for COVID-19. Although the patient was initially discharged with mild monocytosis and no cytopenia, further molecular and flow cytometry testing was performed on the sample with promonocytes.

## 2. Case Report

**Figure 1:**
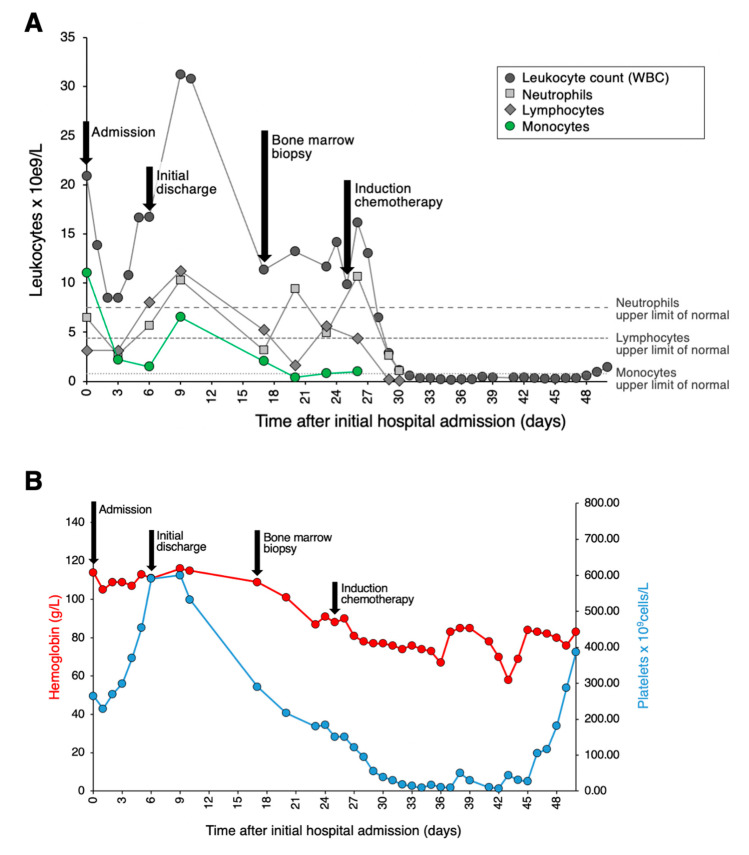
Clinical course and hematological lab values. (**A**) Graph depicting the patient’s clinical course and total leukocyte, neutrophil, lymphocyte, and monocyte counts. Admission to tertiary care is set as Day 0. (**B**) Graph depicting the patient’s clinical course along with hemoglobin and platelet counts.

**Figure 2:**
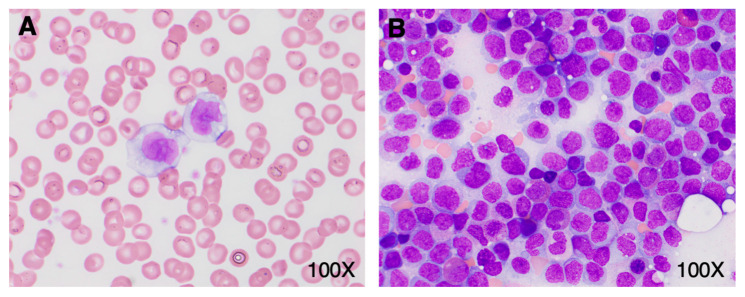
Promonocyte morphology in peripheral blood and bone marrow. (**A**) Peripheral blood smear on admission showing classical promonocyte morphology. (**B**) Bone marrow aspirate (Wright-Giemsa stain) with a large number of promonocytes.

A 41-year-old woman with a history of hypertension, obstructive sleep apnea, ocular rosacea, recurrent urinary tract infections, and splenectomy for benign cysts was admitted to hospital for COVID-19-related pneumonia. She had no history of cytopenia or monocytosis; her complete blood count (CBC) from one year prior showed a minimal decrease in hemoglobin at 118 g/L and a platelet count of 589 × 10^9^/L. She had received one dose of the Pfizer-BioNTech mRNA COVID vaccine 6 weeks prior to hospital admittance. During her course in hospital, she required supplemental oxygen, including OptiFlow as high as 70% FiO_2_, and was treated with dexamethasone, ceftriaxone, azithromycin, and tocilizumab. Her white blood count (WBC) on admission was 20.86 × 10^9^/L with a differential of 11.06 × 10^9^/L monocytes, 6.47 × 10^9^/L neutrophils, and 3.13 × 10^9^/L lymphocytes (Figure 1A). Her levels of hemoglobin and platelets were within the normal range (Figure 1B). A review of her peripheral blood smear showed an erythroblast count of 1/100 WBC and increased monocytes with various degrees of vacuolated cytoplasm. There was minimal dysgranulopoiesis with overall unremarkable red cells and platelets. Notably, half of the monocytes (with an estimated absolute count of about 5.50 × 10^9^/L) exhibited morphologic features of promonocytes, including large cell size, abundant pale blue-to-gray cytoplasm with varying content of fine azurophilic granules with slightly lobulated nuclei, and finely dispersed chromatin (Figure 2A). The monocytes showed strong CD56 expression, heterogeneous expression of CD13, partly upregulated CD16, and only slightly weaker CD14 expression. A small population comprising 0.7% of total viable cells showed CD117 expression with absent CD34. Three days later, the patient’s overall status markedly improved. At this time, the WBC was 8.5 × 10^9^/L with 3.1 × 10^9^/L lymphocytes, 2.71 × 10^9^/L neutrophils, and 2.2 × 10^9^/L monocytes. The patient’s hemoglobin and platelet count showed no significant change. At this time, promonocytes accounted for 20% of the monocytes (with an estimated absolute count of about 0.44 × 10^9^/L). Repeat flow cytometry showed minor changes in the monocyte immunophenotype with decreased monocytes with CD56 and CD16 expression. Again, a small population (0.7% of total) expressing CD117 with absent CD34 was detected. The patient was discharged despite a concern from the laboratory that acute leukemia could be excluded. Close follow up, including molecular studies and cytogenetics, was recommended by a hematopathologist.

Subsequently, molecular testing was expedited and performed on the initial peripheral blood sample containing a large number of promonocytes to further investigate the potential clinical significance of these cells. Targeted next generation sequencing (NGS) revealed a *NPM1* c.863_864insTCTG (W288fs) mutation at a variant allele frequency of 17.7% as well as a *DNMT3A* c.2645G>A (R882H) mutation at a variant allele frequency of 24.8%, two pathogenic aberrations that are highly associated with AML. Indeed, *NPM1* mutation is the most common molecular aberration found in AML. Based on these results, the patient was asked to return to the hospital for a bone marrow biopsy. On the day of the biopsy, the patient’s WBC was 11.33 × 10^9^/L with 5.21 × 10^9^/L lymphocytes, 3.17 × 10^9^/L neutrophils, and 2.04 × 10^9^/L monocytes; hemoglobin was minimally decreased at 109 g/L; and platelets were 219 × 10^9^/L. The peripheral blood smear evaluation showed a leukoerythroblastic picture with persisting promonocytes, mild dysgranulopoiesis, and 2% blasts. In the bone marrow aspirate, there were 75% promonocytes/blasts (Figure 2B). The leukemic population showed expression of CD33 (bright), CD13 (variable), CD11b, CD65, HLA-DR (weak), CD38, CD56, CD36, CD64, CD15, CD4, CD11c, TdT, and MPO (dim). This population was negative for CD123, CD19, CD7, CD16, CD117, CD235a, CD2, CD10, CD71, cCD79a, sCD3, cCD3, and cCD22. Cytogenetic studies showed a normal female karyotype with no numeric or structural clonal alterations. Together, these findings were consistent with the diagnosis of AML. The patient was treated with standard therapy for AML. Namely, she received induction therapy with gemtuzumab ozagamicin, consolidation with high-dose cytarabine and gemtuzumab ozagamicin, and is currently in remission.

## 3. Discussion

Monocyte activation mediated by cytokine release constitutes an important component of an effective immune response to viral infection. Patients with COVID-19 have been found to harbor a unique subset of monocytes with a distinct immunophenotype, namely expression of CD14 and CD16, and FSC-high [8]. Flow cytometric analyses have suggested that the immunophenotype of monocyte subpopulations in COVID-19 is dynamic, with differences in expression of HLA-DR and CD16 that are dependent on the severity and course of infection [9]. These changes may be driven by the atypical cytokine storm that occurs during COVID-19 infection that was recently found to be orchestrated by myeloid cells and neutrophils [10]. While our case demonstrated the emergence of a subpopulation of monocytes expressing partly upregulated CD16 and partly weaker CD14 that were concurrent with the peak of infection, the presence of promonocytes could not be explained by a viral infection and was concerning for a second etiology. As the patient’s viral infection resolved, the expression of CD16 on monocytes decreased. The leukemic population at the time of bone marrow biopsy was CD16-negative. 

COVID-19 infection has been associated with other transient hematological changes, including the presence of blasts in peripheral blood and bone marrow [11] and unusual expression or loss of typical immunological markers in leukemia patients [12,13]. In our case, the presence of an atypical cell population that persisted beyond recovery from a viral infection and that was characterized by flow cytometry, cytogenetics, and molecular assays led to the final diagnosis of AML. Importantly, the first diagnostic clue was the presence of promonocytes—immature precursor cells that should be considered as blast equivalents in any clinical setting, even despite a lack of cytopenia and overall clinical improvement. Furthermore, the identification of a defining genetic abnormality in the same sample provided strong support to perform a bone marrow biopsy. Molecular studies should, therefore, be considered when promonocytes are encountered in the peripheral blood, especially when flow cytometry is not definitive. In centers where molecular studies cannot be expedited, the presence of promonocytes on peripheral blood should prompt the consideration of a bone marrow biopsy to prevent any delay in diagnosis or treatment.

## 4. Conclusions

Highly atypical morphology, including abnormal monocytes, can be encountered in the peripheral blood smear of patients with COVID-19. Nonetheless, promonocytes at time of this report remain specific to myeloid neoplasia. In this case report, the surge in leukemic promonocytes and their retreat during clinical improvement is interpreted as an early presentation of AML due to a secondary COVID-19-induced leukoerythroblastic reaction. While subtle immunophenotypic abnormalities were present initially, the morphological findings in combination with molecular evidence of defining genetic abnormalities were pivotal in reaching the final diagnosis of acute leukemia. 

## Data Availability

The data used and analyzed during the current study are available from the corresponding author on reasonable request.
